# Immunization of a wild koala population with a recombinant Chlamydia pecorum Major Outer Membrane Protein (MOMP) or Polymorphic Membrane Protein (PMP) based vaccine: New insights into immune response, protection and clearance

**DOI:** 10.1371/journal.pone.0178786

**Published:** 2017-06-02

**Authors:** Marion Desclozeaux, Amy Robbins, Martina Jelocnik, Shahneaz Ali Khan, Jon Hanger, Volker Gerdts, Andrew Potter, Adam Polkinghorne, Peter Timms

**Affiliations:** 1 Centre for Animal Health Innovation, Faculty of Science, Health, Education and Engineering, University of the Sunshine coast, Maroochydore DC, Queensland, Australia; 2 Endeavour Veterinary Ecology Pty Ltd, Toorbul, Queensland, Australia; 3 Vaccine and Infectious Disease Organization– International Vaccine Centre, University of Saskatchewan, Saskatoon, Saskatchewan, Canada; Midwestern University, UNITED STATES

## Abstract

We assessed the effects of two different single-dose anti-Chlamydia *pecorum* (*C*. *pecorum*) vaccines (containing either Major Outer Membrane Protein (3MOMP) or Polymorphic Membrane Protein (Pmp) as antigens) on the immune response of a group of wild koalas. Both vaccines elicited a systemic humoral response as seen by the production of anti-chlamydial IgG antibodies in more than 90% of vaccinated koalas. A mucosal immune response was also observed, with an increase in *Chlamydia*-specific mucosal IgG and/or IgA antibodies in some koalas post-vaccination. Both vaccines elicited a cell-mediated immune response as measured by the production of the cytokines IFN-γ and IL-17 post-vaccination. To determine the level of protection provided by the vaccines under natural conditions we assessed *C*. *pecorum* infection loads and chlamydial disease status of all vaccinated koalas pre- and post-vaccination, compared to a non-vaccinated cohort from the same habitat. The MOMP vaccinated koalas that were infected on the day of vaccination showed significant clearance of their infection at 6 months post-vaccination. In contrast, the number of new infections in the PMP vaccine was similar to the control group, with some koalas progressing to disease. Genotyping of the *ompA* gene from the *C*. *pecorum* strains infecting the vaccinated animals, identified genetic variants of *ompA-F* genotype and a new genotype *ompA-O*. We found that those animals that were the least well protected became infected with strains of *C*. *pecorum* not covered by the vaccine. In conclusion, a single dose vaccine formulated with either recombinant PmpG or MOMP can elicit both cell-mediated and humoral (systemic and mucosal) immune responses, with the MOMP vaccine showing clearance of infection in all infected koalas. Although the capability of our vaccines to stimulate an adaptive response and be protective needs to be fully evaluated, this work illustrates the necessity to combine epitopes most relevant to a large panel of variable strains with an efficient adjuvant.

## Introduction

Disease caused by the obligate intracellular bacterial pathogen, *Chlamydia*, is a significant threat to the ongoing survival of the koala. Combined with habitat destruction, motor vehicle injuries and dog attacks, this threat has driven many koala populations in Australia to the point of near extinction in some areas [1, 2].

Amongst the 11 species in the genus *Chlamydia* currently recognised, [3–5], *C*. *pecorum* is an important veterinary pathogen that causes debilitating ocular and urogenital infections in koalas with clinical signs such as conjunctivitis, kerato-conjunctivitis, rhinitis, cystitis, infertility and sterility [2]. To date, once a clinical chlamydial infection is detected in a koala, a 4–6 week course of antibiotic such as chloramphenicol is usually administered to clear the infection. Unfortunately, such practice has negative impacts on koala welfare as each treated animal needs to be kept in captivity for the length of the treatment. A successful anti-chlamydial vaccine would allow better management of the disease in the koala population with minimal impact on koala welfare.

Developing an efficient chlamydial vaccine has proven challenging in all animal species to date, as both an efficient delivery system (adjuvant) and specific immunogenic antigen(s) need to be combined to promote both humoral and cell-mediated immune responses [6–8]. Indeed, upon invasion of the mucosal lining by chlamydia, the innate immune system is activated, followed by induction of the adaptive immunity pathways in order to limit the spread of the infection and protect against recurrent infections. The immune response to chlamydia thus includes production of pro-inflammatory cytokines, followed by maturation of T cells into both CD4 and CD8 T cells (recognizing specific MHC-presented chlamydial antigens) and activation of B cells that will produce specific anti-chlamydial antibodies [6, 8, 9]. The cytokine IFN-γ plays a key role in both the innate and the adaptive immune responses against chlamydial infections by contributing to inhibiting the growth of the bacteria chlamydia and activating the T-cell immune response to ultimately trigger protection against re-infections [10]. Similar to IFN-γ, IL-17 appears elevated in koalas with *C*. *pecorum* disease, compared to healthy infected animals, and previous work with *C*. *muridarum* suggests that IL-17 might play a role in clearing the infection by synergistically working with IFN-γ to inhibit chlamydial growth [11–13]. Finally, murine vaccine studies have suggested that IL-17 is also important for protection against new infections [14]. Therefore, both cytokines IFN-γ and IL-17 seem to play crucial roles in the initiation and establishment of an anti-chlamydia immune response, which one would expect to see similarly modulated by a successful anti-chlamydial vaccine.

A major antigen candidate for a chlamydial vaccine is the chlamydial major outer membrane protein (MOMP). MOMP contains four variable hydrophilic domains exposed to the surface on the outer membrane of chlamydia and allows adhesion to the mannose receptor of the host cell, during the initial phase of infection [15, 16]. MOMP has been used in several clinical trials, in different animals, with encouraging results [17–20], eliciting a T cell-mediated response together with IFN-γ production [17, 21–23]. Other antigenic proteins including polymorphic membrane proteins (Pmps) have also been evaluated in mice [24]. Pmps are a group of membrane bound surface-exposed chlamydial proteins [25]. Pmps contain an auto-transporter adhesion domain important for the initial phase of chlamydial infection by contributing to attachment to the mucosal cell membranes possibly via the epidermal growth factor receptor [26]. Each chlamydial genome encodes a variable number of Pmp proteins differentially expressed throughout the chlamydial developmental cycle. For *C*. *trachomatis*, PmpG is the most immunogenic protein in murine vaccine models [27, 28], while PmpD was identified as very immunogenic for *C*. *abortus* and *C*. *psittaci* [29, 30]. The *C*. *pecorum* genome appears to encode at least nine PmpG family proteins [31]. PmpG1 and to a lesser extent PmpG 9 were under positive selection in koala strains, with immune pressure being a likely driver of this selection in this host [31].

Our previous work in koalas showed that a single dose vaccine formulation with a tri-adjuvant (TriAdj) combined with a cocktail of three MOMP antigens was safe and triggered both humoral and cellular immune responses in healthy, *Chlamydia*-negative, female koalas in captivity, and a small subset of non-infected wild koalas [17, 23]. In the current study, we extended this work with the use of the TriAdj vaccine combined to a new *C*. *pecorum* antigen, PmpG, and compared this newly formulated vaccine with the 3MOMP vaccine.

## Materials and methods

### Cloning, expression and purification of recombinant koala C. pecorum PmpG protein

The adhesin domain (also referred as GGAI domain in the literature) of koala *C*. *pecorum* PmpG (aa 27 to aa 520 of the *PmpG1* gene) was PCR amplified using specific primers, kPmg-F (5’-AATGAGCTCGAGACTATCCCCATCCCATCTAAAAATTTC-3’) kPmg-R (5’-TTAGCGGCCGCTTATTTTCTAAGGTGACTTGCTGATTG-3’), to generate a PCR product with 5’Sac1 and 3’Not1 restriction sites (underlined in the sequences). The Sac1/Not1 double digested PCR product was subsequently cloned into Sac1/Not1 double digested pET28a expression vector (Invitrogen), in frame with the N-terminal poly-histidine (His) tag to produce a final protein of 538 aa, including the 504 aa fragment of kPmpG. The pET-His-kPmpG expression vector was transformed into BL21 (DE3) competent *E*. *coli* cells (Bioline) and grown in LB media with 100 μg/mL ampicillin at 37°C. His-kPpmG expression and purification was conducted as per ‘The Expressionist’ protocol for non-soluble proteins (Qiagen). Briefly, protein expression was induced at OD600 of 0.4–0.6, by adding 1 mM IPTG (Astral Scientific) for 7 h. Cells were harvested by centrifugation and resuspended in lysis buffer I (50 mM phosphate buffer pH 7.0, 8M urea, 50 μL Bacterial Protease Inhibitor Cocktail (Sigma-Aldrich)), and 1 mg/mL lysozyme (Sigma-Aldrich). Cells were lysed by sonication on ice and the clear lysate incubated with TALON affinity resin (Ni-NTA, Qiagen) at RT for 1 h with gentle mixing. After repeated washes (50 mM phosphate buffer pH 7.0, 300 mM NaCl, and 8M urea) of the resin on a gravity column, the His-kPmpG protein (which will be referred as PmpG protein from now on) was eluted with 50 mM phosphate buffer pH 7.0, 300 mM NaCl, 150 mM imidazole and 8 M urea, at pH 5.6, then pH 4.5. Protein was visualized by western blot using anti-His antibody (Life Technologies Australia Pty Ltd) and IRDye^®^ 800CW Donkey anti mouse (LICOR), purity assessed by SDS-PAGE (Bio-rad), and protein concentrations determined with Micro BCA Protein Assay Kit (Pierce). A schematic of the PmpG protein used in the vaccine is provided in S1 Fig.

### Expression and purification of C. pecorum MOMP-A, -F and G recombinant proteins

Koala recombinant proteins from three different serotypes (MOMP-A,—F, and—G) previously described and genotyped by Kollipara *et al*. [32] were purified according to the published protocol of with modifications as follows: (A) cell lysis were performed by sonication on ice with addition of lysozyme; (B) after binding of His-MOMP proteins to the Ni-NTA beads (Qiagen), washes and elution were performed on a gravity column to prevent contamination of the eluate with affinity beads. A schematic of the MOMP-A,—F, and—G proteins used in the vaccine is provided in S1 Fig.

### Vaccine formulation

Endotoxin content was determined for each purified recombinant protein using the LAL Chromogenic Endotoxin Quantitation Kit (Pierce) according to the manufacturer’s protocol. Quantities of endotoxin determined were as follow: 2.63 EU/mL for the 3MOMP proteins, and 0.54 EU/mL for the PmpG protein.

TRiAdj 3MOMP and PmpG vaccines were prepared according to a previously published protocol by Garlapati *et al*. [33]. A final concentration of 150 μg of 3MOMP proteins (50 μg each MOMP protein) or 150 μg of PmpG protein were co-formulated at a ratio 1:2:1 with PCEP (250 μg; poly[di(sodium carboxylatoethylphenoxy)]-phosphazene), IDR1002 (500 μg) and polyI:C (250 μg) in PBS, all provided by VIDO-intervac (University of Saskatchewan, Saskatoon, SK, CA). For each vaccine, 500 μl injection doses were then aliquoted in endotoxin-free sterile glass vials (Thermofisher) surrounded with aluminium foil to protect from the light, and stored at -20°C until injection.

### Experimental groups of koalas, immunization schedule, sampling

For this study, 63 koalas located in the Moreton bay region, Queensland, Australia (and part of the Moreton Bay Rail Link project by the Queensland Government Department of Transport and Main Roads) were selected after thorough veterinary health assessment by experienced wildlife veterinarians. The koalas were enrolled in the trial according to the following criteria: koalas were older than 1 year old (breeding age) and showed no clinical signs of chlamydial disease (chlamydiosis) such as ocular or UGT discharge or severe inflammation, and negative Clearview^®^ Chlamydia test. The selected koalas were randomly assigned into three groups: group ‘3MOMP’ vaccine was vaccinated with the 3MOMP TriAdj vaccine (3MOMP vaccine), group ‘PmpG’ vaccine was vaccinated with the PmpG TriAdj vaccine (PmpG vaccine) and a control group was non-vaccinated. All koalas were sampled prior to immunisation (21 animals in the 3MOMP vaccine cohort, 21 animals in the PmpG vaccine cohort) and then between 5 to 7 months post immunisation (when capture was judged possible and safe–referred to as the” 6-month post-vaccination” time point here). Peripheral blood mononuclear cells (PBMCs) were purified from blood samples according to Mathew *et al* (2013; [34]). Serum was separated from coagulated blood by centrifugation at *1000g* for five min at RT and stored at -20°C for further analysis. Ocular and UGT swabs were collected for mucosal immunity studies in 1.5 mL eppendorf tubes containing 1 mL PBS plus protease inhibitor (ROCHE) and frozen at -20°C. For *C*. *pecorum* infection screening, dry ocular and UGT swabs were collected and stored at -20°C until genomic DNA extraction.

All procedures were approved by the University of the Sunshine Coast (USC) Animal Ethics Committee (Animal ethics number AN/A/13/80) and by the Queensland Government (Scientific Purposes Permit, WISP11532912). The trial was performed under the Australian Pesticides and Veterinary Medicines Authority Permit PER 725.

### Koala C. pecorum-specific IgG and IgA ELISA

ELISAs were performed according to Carey *et al*. [35] with modifications. Briefly, 96-well plates (medium-high binding, SIGMA) were coated overnight at 4°C with His-MOMP, or His-PmpG proteins (1 μg per 50 μl, per well), or 1 μg of semi-purified *C pecorum* strain G EBs (purified according to [35]) in carbonate-bicarbonate buffer. After washing and blocking in 0.05% Tween-PBS (PBS-T) with 5% skim milk, all subsequent incubations were carried out at 37°C for 1 hour separated by 3 washes with PBS-T. Following blocking with milk, 1:3 serially diluted sera were added to the wells. Initial dilution for all sera was 1:100. All swab (mucosal) samples were initially diluted 1:2, then, serially diluted 1:2. For IgG ELISAs, the secondary antibody incubation was performed using sheep anti-koala IgG diluted 1:8000 in PBS-T, followed by a final incubation with HRP-conjugated donkey anti-sheep IgG (1:10000, ABCAM). For IgA ELISAs, the second incubation used a rabbit anti-koala IgA antibody (see IgA Section) at 1:2000 and the final incubation used HRP-conjugated goat anti-rabbit IgG (1:10000, ABCAM). TMB substrate in citrate buffer, prepared as per the manufacturer (Sigma-Aldrich), was added to each well and, after 20 min of incubation at RT, reactions were stopped by adding the same volume of 1M H2SO4. Optical density was determined at 450nm. All samples were tested in duplicate.

End point titres (EPT) were calculated as the inverse of the dilution value at which the tested serum is no longer giving a positive signal using Graph Pad Prism. The cut off values were calculated as the average of the ‘no sample’ control values added to the standard deviation multiplied by two. The ‘no sample’ control corresponded to the addition of PBS-T instead of the first sera or swab sample after milk blocking, all other incubations remaining the same.

### Preparation of rabbit anti-koala IgA

Purified recombinant koala IgA heavy chain constant region was produced and purified by affinity and size exclusion chromatography by Protein Expression Facility (PEF; University of Queensland, Brisbane, Australia) and subsequently used to immunize rabbits. Following serum isolation from two independent rabbits, specific polyclonal anti-IgA antibodies were affinity purified (Mimotopes, The Peptide Company, Victoria, Australia). Purified anti-koala IgA was tested and validated by ELISA and western blot (data not shown). In this study, anti-koala IgA was used at 1/2000 dilution in the ELISA.

### Koala lymphocyte stimulation assay. RNA extraction, reverse transcription and qRT-PCR assays

Experiments to assess gene expression of koala IFN-γ, Il-17 and IL-10 were performed as previously described by Mathew *et al*. [11, 36] with GAPDH as internal control. As such, purified peripheral blood mononuclear cells (PBMCs) from 3MOMP or PmpG vaccinated koala blood samples were diluted to a concentration of 2×10^6^ cells/mL and stimulated for 12 hours with UV inactivated semi-purified *C*. *pecorum* G strain EBs at a final dilution of 1:10. PBMCs were suspended in 1 mL of Trizol reagent (Invitrogen, Australia), RNA extracted and cDNA synthesized. All reactions were carried out in a final volume of 20 μL, containing 5 μL of cDNA sample, 1 μL of 10 μM forward and reverse primers and 1X QuantiTect SYBR^®^ Green PCR mix (Qiagen) as previously described [11, 36]. All samples were tested in duplicates.

### Screening for C. pecorum infection by qPCR

Pre- and post-immunization ocular and urogenital tract (UGT) dry swabs were screened for the presence of *C*. *pecorum* infections by 16S rRNA gene *C*. *pecorum* quantitative PCR modified from Marsh *et al*. [37]. The *C*. *pecorum* 16S 204 bp fragment (RT-Cpec -F: 5'-AGTCGAACGGAATAATGGCT-3', RT-Cpec-R: 5'-CCAACAAGCTGATATCCCAC-3'; IDT) was sub-cloned into pGem-T Easy (Promega) and amplified with M13 universal primers to generate a M13-Cpec-16S fragment of 465 bp. Serial dilutions of the M13-Cpec-16S fragment were used to produce a standard curve by mixing 5 μl of diluted fragment with RT-Cpec-F and RT-Cpec-R primers (1μM final) and 1X QuantiTect SYBR^®^ Green PCR mix (Qiagen) in a final volume reaction of 20 μl. Cycling conditions were 95°C- 15 min, followed by 35 cycles of 94°C- 15 sec, 57°C- 15 sec, 72°C- 30 sec, and a final amplification cycle of 72°C, 10 min. Diluted *C*. *pecorum* G type strain served as a positive control while dH_2_O was used as negative control. A detection level of 100 copies/μL was established and values below this result were reported as ‘Below Detection Level’ (BDL). All samples were tested in duplicate.

### C. pecorum ompA sequencing, alignment and phylogeny

Genetic diversity in the MOMP-encoding *ompA* gene of *C*. *pecorum* strains detected in the vaccinated koalas was determined by amplifying the near full length *ompA* gene (1140 bp) using conventional PCR. Where multiple PCR positive samples were available for a koala, the sample with the highest qPCR load was selected for *ompA* gene sequencing. The primers used in this reaction were *ompA*-F (5′-ATGAAAAAACTCTTAAAATCGG-3′) and *ompA-*R (5′-TTAGAATCTGCATTGAGCAG-3′). PCR conditions were a single cycle of initial denaturation at 95°C for 10 min, 40 cycles of denaturation at 95°C for 30 s, primer annealing at 57°C for 40 s, primer extension at 72°C for 90 s, followed by a final extension at 72°C for 7 min. For koalas presenting a qPCR load less than 3000 16S rDNA copies/μL, a second round of amplification was attempted on the purified product from the first PCR. All *ompA* sequences were determined by Sanger sequencing of the Forward and Reverse *ompA* PCR products (AGRF, Brisbane, Australia).

Phylogenetic analysis on 10 koala *C*. *pecorum omp*A sequences identified in this study was performed with the Geneious 9.1 software (http://www.geneious.com; [38]). Briefly, forward and reverse chromatograms for each *omp*A gene were aligned, and a consensus sequence was obtained and trimmed so that all sequences were of the same length. The obtained *omp*A sequences were aligned using ClustalW (as implemented in Geneious 9.1), and also translated into amino acid sequences and aligned. DnaSp v5.1 [38] was used to analyse sequence polymorphisms such as total number of polymorphic sites and haplotypes, as well as the number of non-synonymous (d_n_) and synonymous (d_s_) substitutions per site (Jukes-Cantor corrected). A mid-point rooted Bayesian phylogenetic tree for the 24 *omp*A sequences, including the 10 koala *C*. *pecorum omp*A sequences generated in this study, and 13 previously described koala *C*. *pecorum omp*A sequences [39], and bovine *C*. *pecorum* E58 *omp*A sequence (accession number CP002608), was constructed with MrBayes (as implemented in Geneious 9.1). Parameters included HKY +I+G model with four MCMC chains with 1 000 000 generations, with sub-sampling frequency of 1 000, and 10 000 trees discarded as burn-in. The bovine *C*. *pecorum* E58 *omp*A sequence was used as an out-group. The 10 koala *C*. *pecorum ompA* sequences (M1, M11, M8, M14, M17, C11, C12, C13, P4, P8) from this study are available in Genbank under accession numbers KX388198 (M1), KX388199 (C13), KX388200 (C12), KX388201 (P8), KX388202 (M11), KX388203 (C11), KX388204 (M8), KX388205 (P4), KX388206 (M14), KX388207 (M17).

### Statistics

All statistical analyses were performed using GraphPad Prism version 5 (GraphPad Software, LaJolla, CA, USA) and IBM SBSS statistics 22. All IgG ELISA data and cytokine levels presented include the mean of 21 koalas for each cohort. Statistical significance of these data pre- and post-vaccination were determined by using Wilcoxon signed rank tests with the p values set at *p<0.05, **p<0.01, ***p<0.005, ****p<0.001. To evaluate the contingency between decrease in chlamydial load and vaccination, and development of chlamydiosis and vaccination, we used Fisher’s exact test with the same p value setting as previously stated.

## Results

### Systemic antibody responses post-vaccination with the vaccines

**Fig 1:**
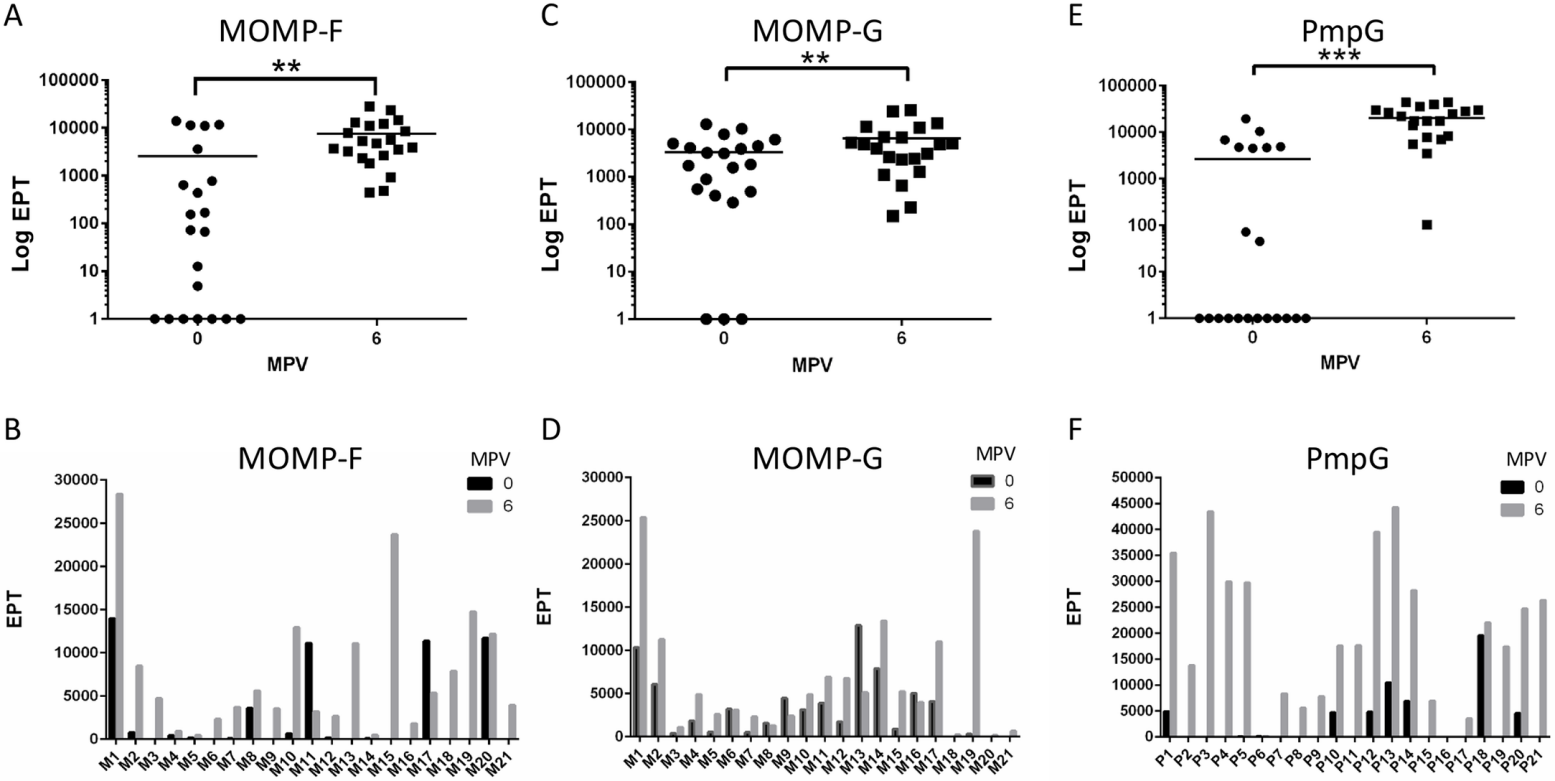
Systemic antibody responses post-vaccination with the 3MOMP and Pmp vaccines. End point titre (EPT) of anti-MOMP and anti-PmpG IgGs in sera from pre-vaccination and 6 months post-vaccination koalas determined by ELISA. IgG titres against purified recombinant MOMP-F and MOMP-G proteins are shown for the 3MOMP vaccine for the whole cohort (panels A and C). For PmpG vaccine, recombinant purified PmpG protein was tested (panels E and F) with sera from PmpG vaccine koalas. Panels A, C, and E show a representation of the results as Log EPT for the whole 3MOMP vaccine or PmpG vaccine cohorts, pre- and 6 months post-vaccination (mean values are indicated). Panels B, D, F show the EPT values obtained for each koala in each vaccine cohort. MPV stands for Months Post-Vaccination. P values were calculated using Wilcoxon matched-pairs signed rank t-test are documented in the results section.

Antigen-specific antibody responses from animals in both vaccine cohorts (3MOMP and PmpG) were evaluated by ELISA, using purified recombinant MOMP or PmpG proteins (S1 Fig), and sera collected from koalas at the pre-vaccination and 6 months post-vaccination time points (MPV; Fig 1). For the 3MOMP vaccine, each MOMP antigen was evaluated separately. The 3MOMP vaccine showed a significant increase in IgG end-point titre (EPT) for both MOMP-G (P = 0.009) and MOMP-F (P = 0.0016) antigens (Fig 1A and 1C). However, we detected low EPT values and no significant difference in serum IgG for anti-MOMP-A antibodies pre- and post-vaccination. All three recombinant MOMP proteins were produced and purified using a similar protocol. When examined individually, koalas vaccinated with 3MOMP showed a wide range of antibody response, with 57% koalas (12/21) exhibiting a modest 2-fold increase in EPT 6 months post-vaccination for MOMP-G and 67% koalas (14/21) exhibiting a 10-fold increase in EPT for MOMP–F (Fig 1D). Interestingly, 47% of koalas (10/21) showed an increased EPT for both MOMP-F and–G (see koala M1 and M19 for example), while 38% only seroconverted for one of the antigens. PmpG vaccinated koalas also showed a significant increase in EPT 6 months post-vaccination (Fig 1E; p<0.0001), with 57% of koalas (12/21) giving a 10-fold increase in EPT (Fig 1F). Together, the data show that both 3MOMP and PmpG vaccines elicited an antibody response in a total of 19 koalas out of 21 (90.5%) or 21 koalas out of 21 (100%) respectively, with PmpG vaccine exhibiting the highest EPT values overall.

**Fig 2:**
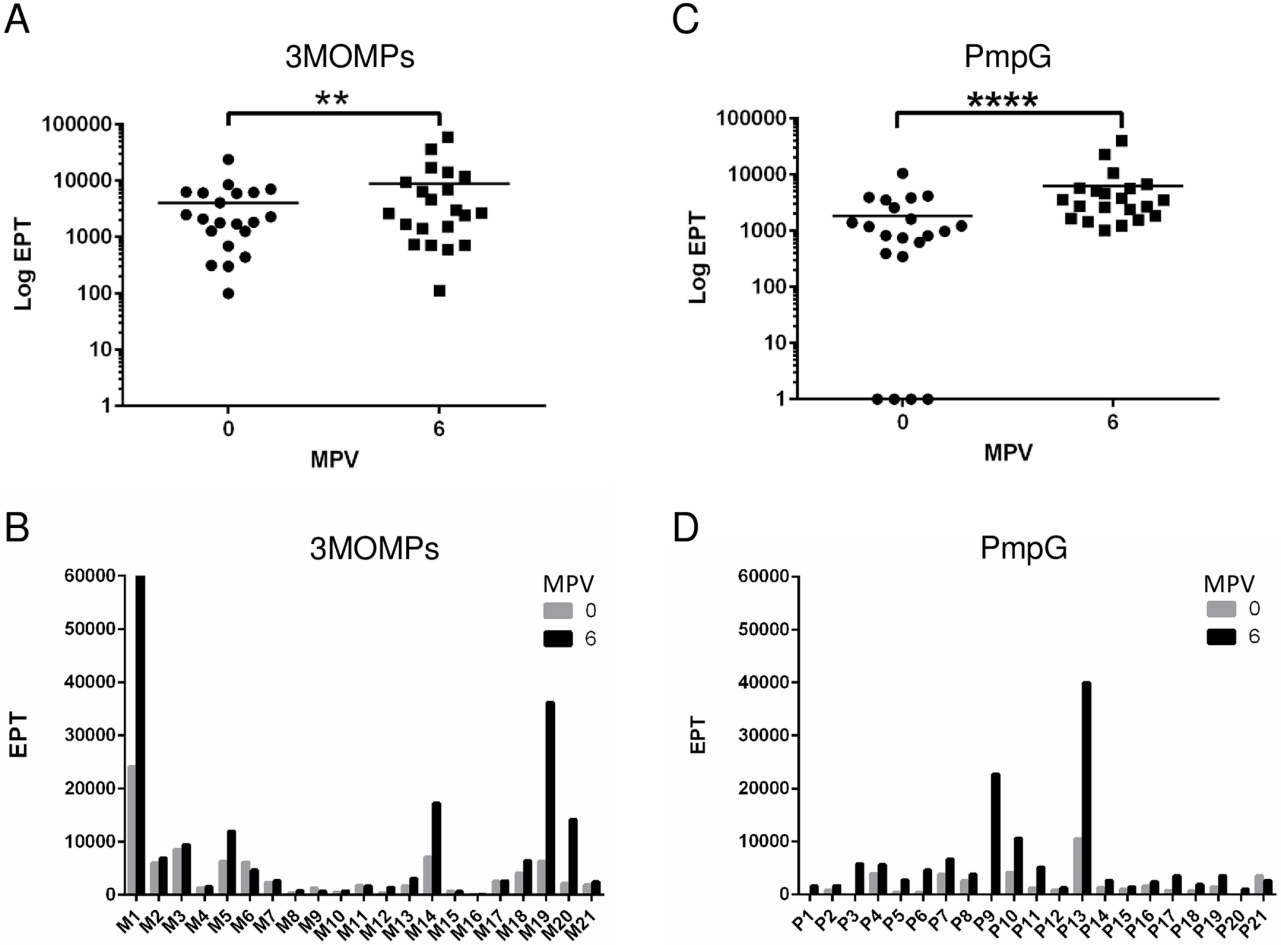
Systemic IgG antibody responses to *C*. *pecorum* EBs post-vaccination with the 3MOMP and Pmp vaccines. Specific IgG antibody response to whole *Chlamydia pecorum* serovar G elementary bodies (EBs) in sera following immunization with 3MOMP and PmpG vaccines. Serums from vaccination day or 6 months post-vaccination were assayed for antigen-specific IgG responses by ELISA using whole EBs. (A) and (C) represent EPT and statistics for 3MOMP and PmpG vaccinated cohorts respectively. (B) and (D) show the EPT obtained for each koala in each cohort. MPV stands for Months Post-Vaccination. P values (Significance was analysed using Wilcoxon matched-pairs signed rank t-test) are documented in the results section.

Reactivity of 3MOMP and PmpG immunized koala sera to whole inactivated *C*. *pecorum G* EBs was also assessed using ELISA (Fig 2). Both vaccine cohorts developed a significant increase in IgG titre to *C*. *pecorum*, 6 months post-vaccination (Fig 2A and 2C; 3MOMP vaccine: p = 0.019; PmpG vaccine: p<0.0001). In the 3MOMP vaccinated cohort, 33% koalas (7/21) developed a 2-fold or higher increase in EPT post-vaccination (Fig 2B). The PmpG vaccine cohort showed a higher ratio with 91.9% koalas (13/21) exhibiting a 10-fold or more increase in EPT values after vaccination (Fig 2D). Altogether, these results demonstrated that both 3MOMP and PmpG vaccines can trigger a humoral immune response in more than 90% of vaccinated koalas and that the IgG antibodies produced as a result of both vaccinations can recognize native epitopes present on intact *C*. *pecorum* EBs.

### Mucosal immune responses in vaccinated koalas

**Fig 3:**
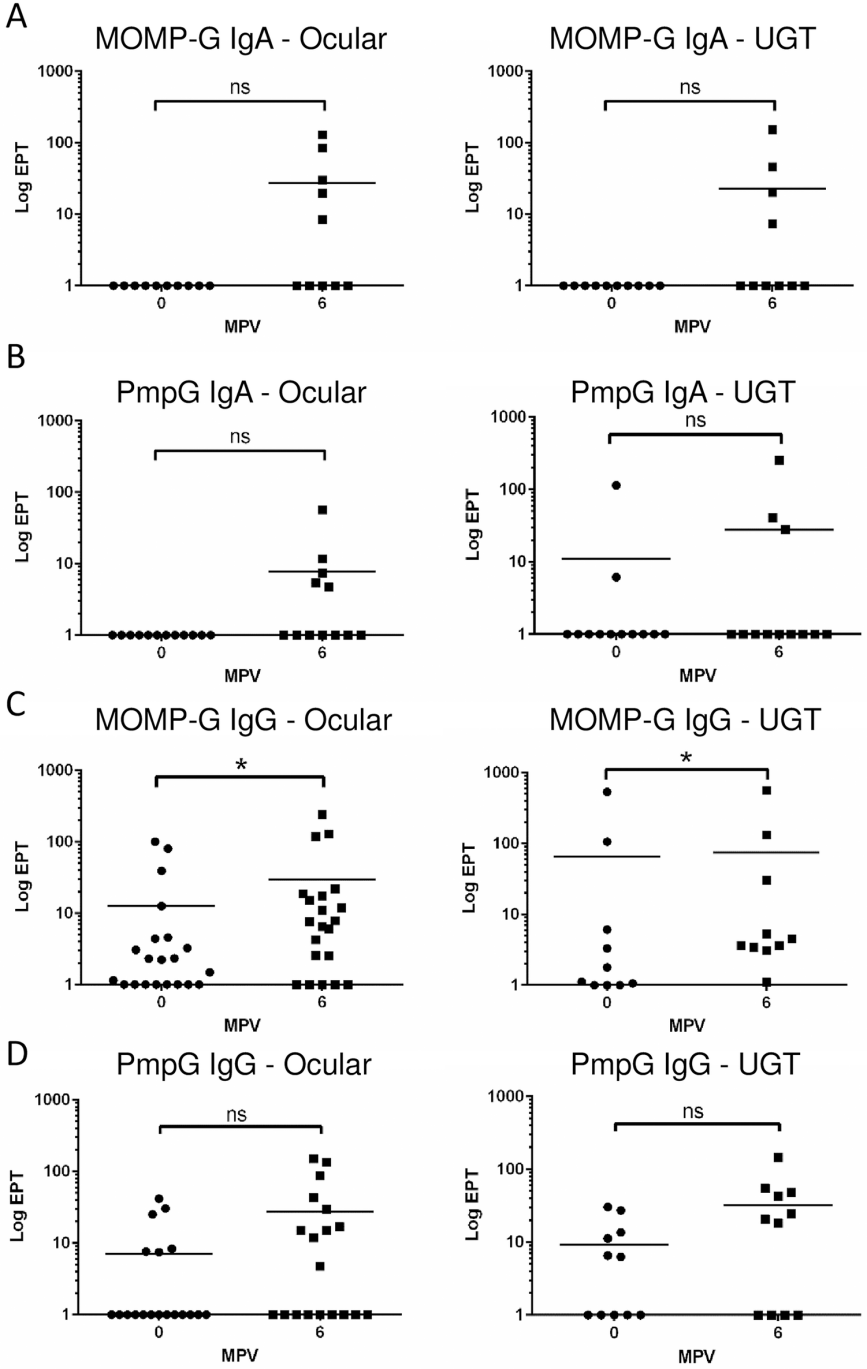
Mucosal specific IgG and IgA antibody response to MOMP and PmpG vaccines. Mucosal specific IgG and IgA antibody response to MOMP and PmpG antigens in ocular and UGT swabs samples following immunization with 3MOMP and PmpG vaccines. Swabs from vaccination day or 6 months post-vaccination (ocular and UGT) were assayed for antigen-specific IgG responses by ELISA using purified recombinant proteins MOMP and PmpG. Right and left panels represent the EPT values and statistics obtained for MOMP-specific IgAs at the ocular and UGT sites respectively, for the 3MOMP vaccinated cohort. Fig 3 B represents the EPT values and statistics obtained for PmpG-specific IgAs at the ocular or UGT sites for the PmpG vaccinated cohort. Panels (C) and (D) show the IgG EPT obtained at either mucosal site (ocular and UGT, left and right panels respectively) in 3MOMP or PmpG vaccine cohort respectively. MPV stands for Months Post-Vaccination. P values obtained using Wilcoxon matched-pairs signed rank t-test are documented in the results section.

To assess the induction of mucosal immunity by both 3MOMP and PmpG vaccines, we measured the IgG and IgA antibody titres in ocular and urogenital (UGT) swabs, pre- and post-immunization, using ELISA assays with recombinant MOMP and PmpG purified proteins (Fig 3). Despite large variations of EPT values amongst koalas within both cohorts, a mucosal immune response was observed with both vaccines in a sub-group of the vaccinated koalas. Indeed, the 3MOMP vaccine increased the EPT titre for anti-MOMP IgA by 10 to 100-fold, at both ocular (5/10 koalas, p = 0.0625) and UGT sites (4/10 koalas; p = 0.125; Fig 3A). Similarly, the PmpG vaccine triggered a 10 to 100-fold increase in EPT values for IgA antibodies at the UGT (4/10 koalas; p = 0.25) or the ocular sites (5/10 koalas; p = 0.0625) post-vaccination (Fig 3B). Evaluation of an IgG response at the mucosal sites of koalas vaccinated with the 3MOMP vaccine showed a more modest but significant 2-fold increase or higher in mucosal anti-MOMP IgG in both ocular (57.1%; 12/21) and UGT samples (50%; 5/10) 6 months post-vaccination (Fig 3C, P = 0.009 and P = 0.0195 respectively). In 10 koalas where both ocular and UGT samples were measured, 50% presented an increase at both sites (data not shown). For the PmpG vaccine, although statistically non-significant, 42% (8/19) and 63% (7/11) of koalas showed a 2-fold or higher increase in either ocular or UGT mucosal IgG recognising recombinant PmpG 6 months post-vaccination (Fig 3D; P = 0.0681 and P = 0.0781 respectively). Overall, our data demonstrated that both 3MOMP and PmpG vaccines did elicit a mucosal response in some koalas by triggering production of 3MOMP and PmpG-specific IgG and IgA antibodies at ocular and UGT sites.

### Cytokine expression in vaccinated animals

**Fig 4:**
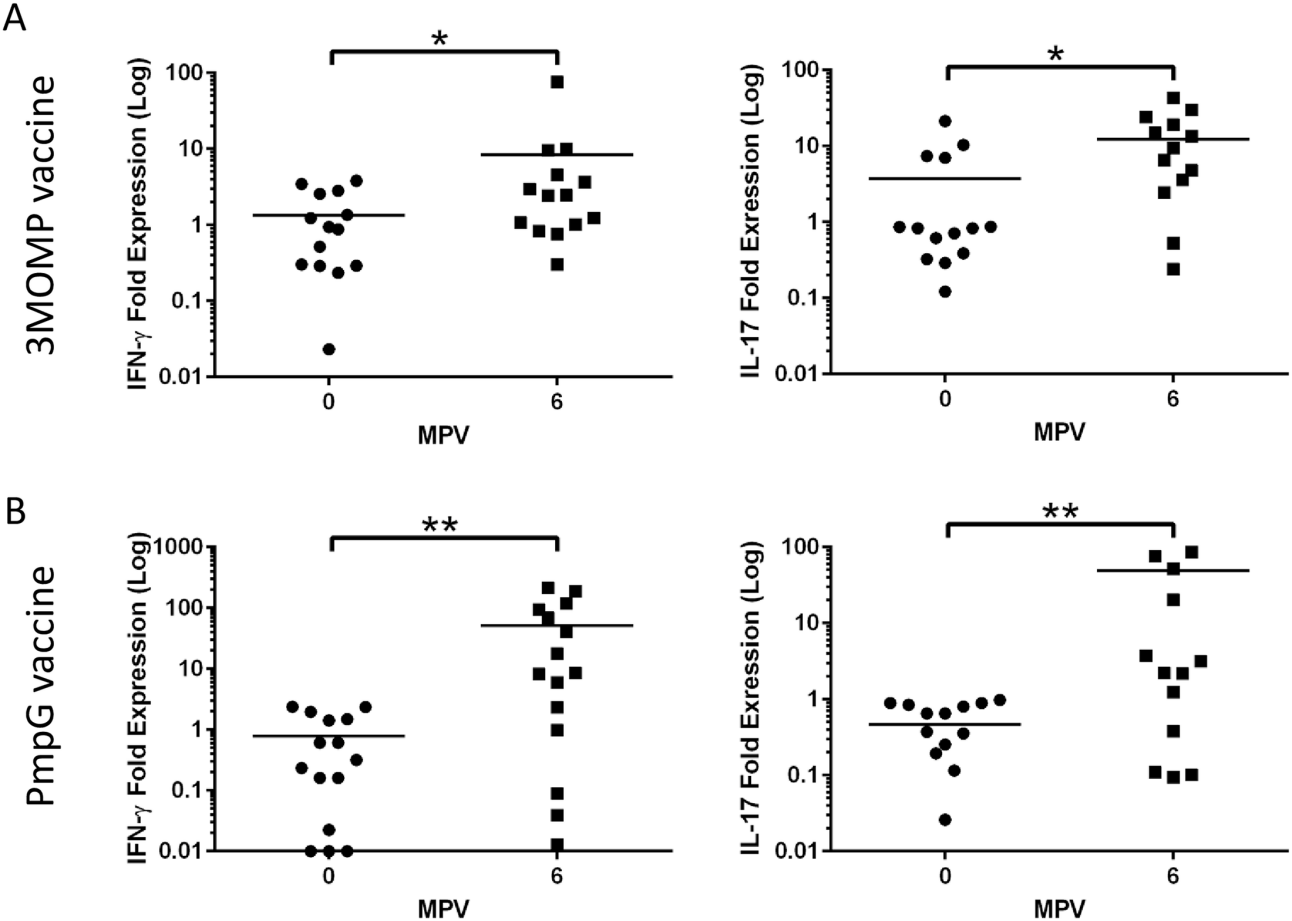
IFN-γ and IL-17 expression in vaccinated koalas. Gene expression of IFN-γ and IL-17 cytokines pre and post immunization. Gene expression of cytokines IFN-γ and IL17 were quantified using RT-qPCR assays on PBMCs from vaccinated koalas, stimulated with *C*. *pecorum* EBs. Data represent the mean-fold change expression of either 3MOMP (panel A) or PmpG (panel B) vaccinated koalas relative to house-keeping gene GAPDH, for IFN-γ and IL17 respectively. Significance was analysed using Wilcoxon matched-pairs signed rank t-test.

Previous research suggests that the host defence mechanisms to chlamydial infections involves secretion of IFN-γ and IL-17 [9]. We therefore measured the chlamydia-specific response of these two cytokines pre- and post-vaccination with 3MOMP and PmpG vaccines using RT-qPCR on purified circulating PBMCs, after *in vitro* stimulation with UV inactivated EBs (Fig 4). Both 3MOMP and PmpG vaccines elicited a significant increase in IFN-γ and IL-17 in EB-stimulated PBMCs (Fig 3A and 3B respectively). For koalas vaccinated with 3MOMP, 57.1% (8/14) showed an increase in IFN-γ P = 0.0495) and 78.6% (11/14) showed an increase in IL-17 (p = 0.017) post-vaccination (Fig 4A, panel right and left respectively). A total of 8 koalas out of 14 exhibited a 2-fold or more increase in IFN-γ gene expression post-vaccination, while IL-17 gene expression increased more than 2-fold in 9/14 koalas post-vaccination. Overall, 50% of 3MOMP vaccinated koalas displayed a 2-fold or more increase in both IL17 and IFN-γ. Similarly, 86.6% (13/15; p = 0.015) of the PmpG vaccinated koalas exhibited an increase in IFN-γ and 100% (15/15, p = 0.0015) in IL-17, post-vaccination (Fig 4B, panels left and right respectively). IFN-γ expression increased by more than 2-fold in 66.7% of koalas post-vaccination while IL-17 expression increased by 2-fold or more in 80% of the vaccinated koalas. Similar to 3MOMP vaccinated koalas, 50% of PmpG vaccinated koalas displayed a 2-fold or more increase in both IFN-γ and IL-17 expression post-vaccination.

Interestingly, for both 3MOMP and PmpG vaccines, we did not observe any correlation between the antibody response and cytokine production, as some koalas exhibiting a strong humoral response showed a poor cell-mediated response and vice versa (data not shown). However, post-vaccination expression levels of IFN-γ and IL -17 were significantly higher in some of the PmpG vaccinated koalas compared to the 3MOMP vaccinated cohort. Indeed, with the PmpG vaccine, 33% koalas had a 200-fold or more increase in IL-17 and 33% koalas had a 90-fold or more increase in IFN-γ, and only one koala showed such a strong increase in both cytokines. Although biological variation amongst koalas was observed, these results showed that both 3MOMP and PmpG vaccines induce production of both host defence IFN-γ and IL-17 cytokines, with the PmpG vaccine triggering a more robust increase in expression of both cytokines compared to the 3MOMP vaccine.

### Chlamydia infectious loads in koalas, pre- and post-vaccination

Although all of the koalas in our study group were clinically healthy at the time of vaccination, we assessed the presence of *C*. *pecorum* in ocular and UGT swabs for each koala pre- and post-vaccination using qPCR (Table 1). The qPCR loads were compared with a control group of 21 koalas from the same geographical area, which received no vaccination. At the time of vaccination, both non-vaccinated (Table 1) and 3MOMP (Table 2) vaccinated cohorts had koalas with a chlamydial infection but no sign of clinical disease: 9.5% non-vaccinated koalas (2/21; C5, C12), and 28.6% of 3MOMP koalas (6/21; M2, M8, M10, M11, M14 ad M17). The PmpG (Table 3) cohort had no koala (0/21) presenting a chlamydial infection at the time of vaccination. We then determined the chlamydial infection load at the 6-month time point in all cohorts. For the non-vaccinated group, four koalas out of 21 (19%) had a load that increased at the 6-month time point compared to the time point 0 [2/21 koalas increased at both ocular and UGT sites (C11 and C13), and three new infections (C6, C11 and C13; qPCR load were below detection level (BDL) at time point 0)]. During the 6-month trial time, koala C12 progressed to chlamydiosis and was treated with antibiotics, clearing the chlamydial infection as shown by a below detection level qPCR result at the 6-month time point (Table 1). Koalas C11 and C13 developed chlamydiosis by the 6-month time point, accompanied by a high qPCR load at both sampling sites (Table 1). In the 3MOMP vaccine cohort, while 6 koalas had a positive load at the time of vaccination, all loads were decreased 6 months post-vaccination (6/21 koalas; 4 ocular sites, 4 UGT sites). This was significantly different to the non-vaccinated group where no decrease in chlamydial load was observed in infected koalas (p = 0.048). Importantly, we observed no occurrence of new infections in the 3MOMP vaccine cohort as opposed to the non-vaccinated group that showed three new infections (Table 2). In the PmpG vaccine cohort, 3 koalas (P8, P9 and P11) showed an increase in *Chlamydia* load post-vaccination (3/21 koalas; 2 ocular sites, 2 UGT sites), corresponding to 3 new infections (Table 3). The incidence of new infections in the PmpG vaccine cohort was similar to the non-vaccinated cohort (3/21 koalas newly infected in each cohort). During the 6-months of our trial, no koala in the vaccinated cohorts were reported with chlamydial disease.

**Table 1:** Chlamydia infection in the non-vaccinated cohort.

	Non -Vaccinated					
	Ocular Loads		UGT Loads		Chlamydiosis during the 6-month trial	Chlamydiosis post the 6-month trial
	Months post-vaccine		Months post-vaccine			
	0	6	0	6		
C1	BDL	BDL	BDL	BDL	N	N
C2	BDL	BDL	BDL	BDL	N	Y
C3	BDL	BDL	BDL	BDL	N	N
C4	BDL	BDL	BDL	BDL	N	N
C5	BDL	BDL	176	242	N	N
C6	BDL	518	BDL	BDL	N	N
C7	BDL	BDL	BDL	BDL	N	N
C8	BDL	BDL	BDL	BDL	N	N
C9	BDL	BDL	BDL	BDL	N	N
C10	BDL	BDL	BDL	BDL	N	N
C11	BDL	1212	BDL	10800	Y	N
C12	BDL	BDL	439	BDL	Y	N
C13	BDL	25964	BDL	2552	Y	N
C14	BDL	BDL	BDL	BDL	N	N
C15	BDL	BDL	BDL	BDL	N	N
C16	BDL	BDL	BDL	BDL	N	N
C17	BDL	BDL	BDL	BDL	N	N
C18	BDL	BDL	BDL	BDL	N	N
C19	BDL	BDL	BDL	BDL	N	Y
C20	BDL	BDL	BDL	BDL	N	N
C21	BDL	BDL	BDL	BDL	N	N

Infection with *C*. *pecorum* was assessed in the non-vaccinated control cohort using our *C*. *pecorum* 16S qPCR assay. Loads obtained are indicated for each koala in copies/μL of sample, pre and post-vaccination. BDL stands for Below Detection Level, and corresponds to <100 copies/ μL. Clinical disease status were recorded for each koala whether it occurred during the trial or during the 3 months past the trial period.

**Table 2:** Chlamydia infection in the 3MOMP vaccine cohort.

	3MOMP Vaccine					
	Ocular Loads		UGT Loads		Chlamydiosis during the 6-month trial	Chlamydiosis post the 6-month trial
	Months post-vaccine		Months post-vaccine			
	0	6	0	6		
M1	BDL	BDL	BDL	BDL	N	Y
M2	224	BDL	BDL	BDL	N	N
M3	BDL	BDL	BDL	BDL	N	N
M4	BDL	BDL	BDL	BDL	N	N
M5	BDL	BDL	BDL	BDL	N	N
M6	BDL	BDL	BDL	BDL	N	N
M7	BDL	BDL	BDL	BDL	N	N
M8	BDL	BDL	337	278	N	N
M9	BDL	BDL	BDL	BDL	N	N
M10	BDL	BDL	228	BDL	N	N
M11	590	BDL	186	BDL	N	N
M12	BDL	BDL	BDL	BDL	N	Y
M13	BDL	BDL	BDL	BDL	N	N
M14	219	BDL	284	BDL	N	N
M15	BDL	BDL	BDL	BDL	N	N
M16	BDL	BDL	BDL	BDL	N	N
M17	193	BDL	BDL	BDL	N	N
M18	BDL	BDL	BDL	BDL	N	Y
M19	BDL	BDL	BDL	BDL	N	N
M20	BDL	BDL	BDL	BDL	N	N
M21	BDL	BDL	BDL	BDL	N	N

Infection with *C*. *pecorum* was assessed in the 3MOMP vaccine cohort using our *C*. *pecorum* 16S qPCR assay. Loads obtained are indicated for each koala in copies/μL of sample, pre and post-vaccination. BDL stands for Below Detection Level, and corresponds to <100 copies/ μL. Clinical disease status were recorded for each koala whether it occurred during the trial or during the 3 months past the trial period.

**Table 3:** Chlamydia infection in the PmpG vaccine cohort.

	PmpG Vaccine					
	Ocular Loads		UGT Loads		Chlamydiosis during the 6-month trial	Chlamydiosis post the 6-month trial
	Months post-vaccine		Months post-vaccine			
	0	6	0	6		
P1	BDL	BDL	BDL	BDL	N	N
P2	BDL	BDL	BDL	BDL	N	N
P3	BDL	BDL	BDL	BDL	N	N
P4	BDL	BDL	BDL	BDL	N	Y
P5	BDL	BDL	BDL	BDL	N	N
P6	BDL	BDL	BDL	BDL	N	N
P7	BDL	BDL	BDL	BDL	N	N
P8	BDL	1292	BDL	2290	N	N
P9	BDL	BDL	BDL	230	N	N
P10	BDL	BDL	BDL	BDL	N	N
P11	BDL	167	BDL	346	N	N
P12	BDL	BDL	BDL	BDL	N	Y
P13	BDL	BDL	BDL	BDL	N	N
P14	BDL	BDL	BDL	BDL	N	N
P15	BDL	BDL	BDL	BDL	N	N
P16	BDL	BDL	BDL	BDL	N	N
P17	BDL	BDL	BDL	BDL	N	N
P18	BDL	BDL	BDL	BDL	N	N
P19	BDL	BDL	BDL	BDL	N	N
P20	BDL	BDL	BDL	BDL	N	N
P21	BDL	BDL	BDL	BDL	N	N

Infection with *C*. *pecorum* was assessed in the PmpG vaccine cohort using our *C*. *pecorum* 16S qPCR assay. Loads obtained are indicated for each koala in copies/μL of sample, pre and post-vaccination. BDL stands for Below Detection Level, and corresponds to <100 copies/μL. Clinical disease status were recorded for each koala whether it occurred during the trial or during the 3 months past the trial period.

However, while our main assessment period for the vaccine was 6 months post-vaccination, we were able to make additional clinical observations up to 9 months post-vaccination (Tables 1–3, right column in table). At that time, an additional number of koalas developed chlamydiosis (UGT site) in each cohort: two koalas from the non-vaccinated group, three koalas from the 3MOMP vaccine group and two koalas from the PmpG vaccine group. None of these koalas had shown a positive *C*. *pecorum* qPCR load previously.

Altogether, our data showed that the 3MOMP and PmpG vaccines have different outcomes regarding protection and clearance of chlamydial infections. New infections were observed in both non-vaccinated and PmpG cohorts at both ocular and UGT sites, suggesting that the PmpG vaccine failed to provide adequate protection against new infections in two animals. In contrast, the 3MOMP vaccine seemed to be responsible for the clearing of six existing infections, and the absence of new infections. This vaccine might be efficient in clearing and possibly protection against chlamydia infections. However, two koalas in the non-vaccinated group and PmpG vaccine group, and three in the 3MOMP vaccine group developed new infections leading to chlamydiosis between the 7^th^ and 9^th^ months post-vaccination suggesting that the immune response triggered by the 3MOMP vaccine is short lasting.

### Chlamydial clearance, mucosal IgA, Genotyping of C. pecorum strains infecting koalas in the trial

**Fig 5:**
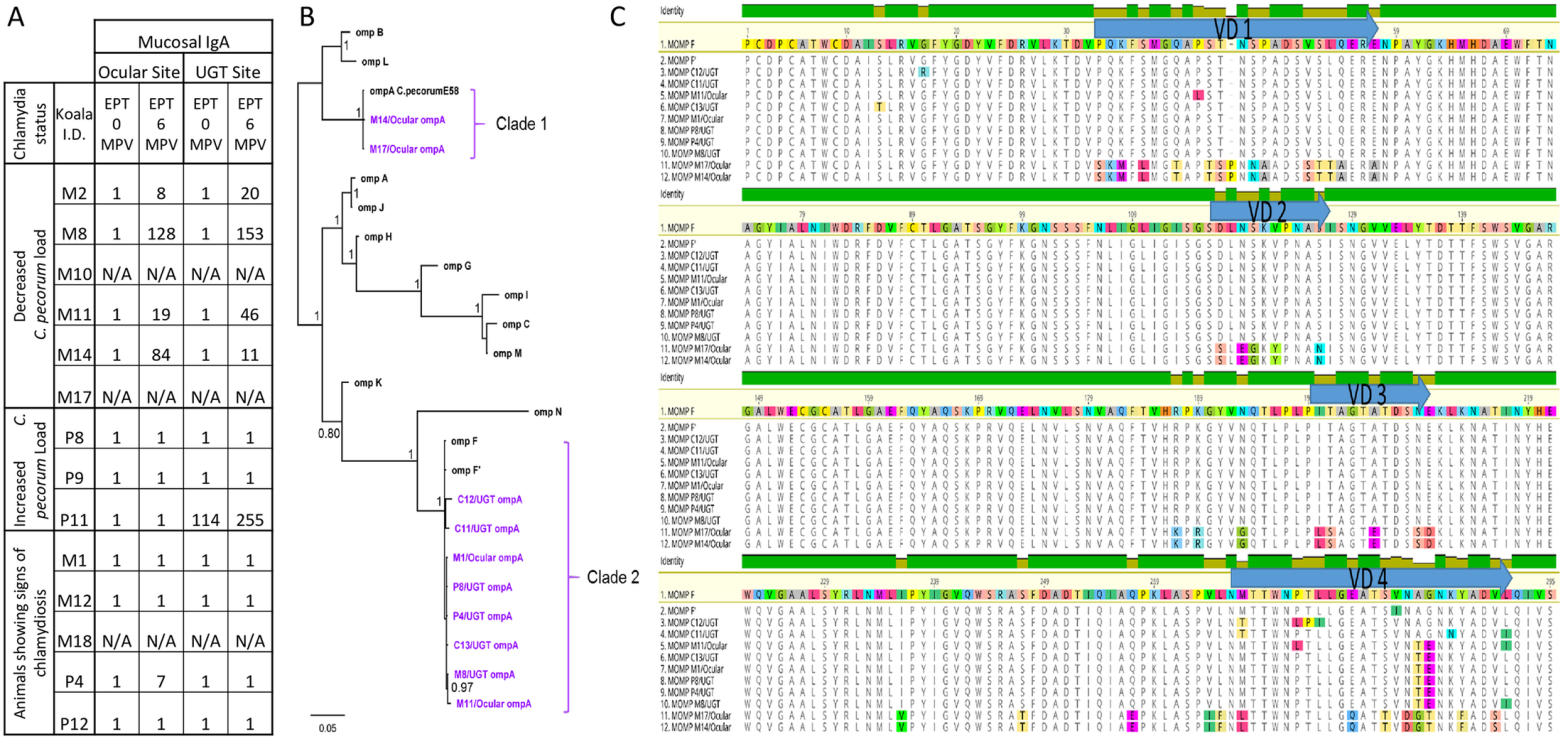
Comparison of chlamydia and disease status with mucosal IgA, alignment and phylogenetic analysis of the new *ompA* genotypes in infected koalas. (A) Infection and disease status was assessed and compared with mucosal IgA in koalas pre- and post-vaccination. (B) Alignment of the new MOMP variants from strains infecting the koalas in the present vaccine trial including the previously described *ompA* F and F’ subtypes. (C) Bayesian phylogenetic analysis of the 24 *omp*A sequences, including the 10 koala *C*. *pecorum omp*A sequences generated in this study, and 13 previously described koala *C*. *pecorum omp*A genotypes. The *C*. *pecorum* E58 *omp*A sequence was used as an out-group. Posterior probabilities are displayed in the tree nodes.

Although we observed that vaccination with both 3MOMP and PmpG vaccines elicited a humoral and cell-mediated immune response, the outcomes of chlamydial clearance and protection against new infections differed between the vaccines. We hypothesized that such differences might be due to (a) koala variations in the immune response to vaccination, or (b) that *C*. *pecorum* strains with genetically variable MOMP and PmpG proteins to those included in the vaccines might be circulating in our wild koala population. We first compared the immunologic characteristics of each infected and diseased koala (data not shown). Regarding the IgG or cell-mediated factors, we could not identify any consistent pattern that the presence or absence of one or more immune factor could be responsible for the lack of protection in the vaccinated koalas or koalas that developed chlamydial disease 9 months post-vaccination. However, when the chlamydia levels and disease status post-vaccination were compared with the mucosal IgA production in each koala, we identified that all koalas with a decreased *C*.*pecorum* load post-vaccination also produced IgA with EPT values between 8<EPT<153 (Fig 5A). The koalas that had an increased *C*. *pecorum* load or were found diseased post-vaccination showed no sign of IgA production in response to vaccination, except for one koala (P11) with a high level of anti PmpG-IgA pre- and post-vaccination. Therefore, this data suggests that production of anti-chlamydial IgA in response to 3MOMP vaccination might be associated with a decrease in *C*. *pecorum* load in infected animals, although further studies would be required.

We then determined which *C*. *pecorum* strains were present in the koalas in this trial population by sequencing the near-full length *ompA* gene for each infected koala. Previous work suggested that the dominant *ompA* genotypes carried by strains circulating in South East Queensland are *C*. *pecorum* F and G [39]. Our vaccine contained recombinant protein made from the three C.pecorum strains, A, F and G. We attempted to perform PCR-amplification and sequencing of the *ompA* gene for each positive koala sample (previously shown in Table 1) but were only able to obtain a PCR product and reliable sequence data for 10/21 koala samples. The sequenced 900bp *ompA* fragments were compared with all other *ompA* genes from all strain types and subtypes previously identified in koalas, and also *ompA* from a reference strain of *C*. *pecorum* identified in Australian livestock, E58. Out of the 10 *ompA* sequences analysed from koalas in our study population, we identified 8 different ompA types. When translated into the corresponding MOMP proteins, alignment with MOMP-F and -F’ clearly showed a majority of non-synonymous mutations in the variable domain 4 (VD4) of the MOMP proteins (Fig 5B). Surprisingly, the *ompA* sequence from two koalas (M14 and M17) showed a significant diversity compared to the *ompA* sequences from the other koalas, suggesting that they were infected with a different chlamydial strain. Phylogenetic analysis revealed that the *C*. *pecorum* koala strains from this population segregated in two distinct, well-supported clades (Fig 5C). Both koalas, M17 and M14, had a *C*. *pecorum* strain distinct from the strains detected in other koalas, which clustered in a clade together with the livestock *C*. *pecorum* E58 strain (Clade 1). M17 and M14 *C*. *pecorum* strains had one synonymous SNP, with M14 identical to the E58 strain. The rest of the isolates all segregated to form Clade 2 together with *C*. *pecorum* F strain and F’ subtype. A difference of 12 nucleotides (nt) in the *ompA* gene (1% of the gene) has been proposed to define a new strain (39). As the differences in these *ompA* genes fluctuated from 8 to 10 nt, we concluded that the koalas in our population were infected with variants of the F chlamydial strain. However, the difference between each koala *ompA* sequences previously reported and the M17 and M14 *ompA* sequences varied from 84 to 175 nt, representing more than 7% divergence. We thus propose that M17 and M14 constitute a new koala *ompA* genotype O (ompA-O), based on the current naming system [31, 40].

## Discussion

In this study, we analysed the immune responses and protective effects of two different anti-chlamydial vaccines delivered as a single dose in a wild population of koalas at 6 months post-vaccination. We first demonstrated that both vaccines elicited a humoral response by inducing the production of anti-chlamydial IgG antibodies in more than 90% of vaccinated koalas. This systemic response to vaccination coincided with a mucosal response observed in some koalas that showed an increase in IgG and/or IgA post-vaccination at ocular and UGT sites. Second, both vaccines were capable of eliciting a cell-mediated immune response via increased production of the two key cytokines, IFN-γ and IL-17. However, both vaccines displayed some differences in their ability to trigger the expected immune response: 3MOMP was a better stimulant of the mucosal immune response, while PmpG resulted in the production of a higher level of cytokines. Third, we assessed the chlamydial loads of all vaccinated koalas pre- and post-vaccination, compared to a non-vaccinated cohort from the same geographical region. The 3MOMP vaccinated koalas that were infected on the day of vaccination showed a clearance of their infection by 6 months post-vaccination, compared to the non-vaccinated infected koalas that did not clear their infection and some of which also progressed to disease. Finally, we identified genetic variants of *C*. *pecorum* in the geographical region of this koala population. These strains had non-synonymous mutations in the immunogenic domains of MOMP. These genetic variations might explain differential outcome of the vaccines on protection and clearing of the disease, as they were different to the variants used in our vaccines.

We used two different antigens, MOMP and PmpG, and found no major differences in the resulting immune responses observed between both vaccinated cohorts. Interestingly, some animals showed a high anti-Chlamydia IgG titre prior to vaccination which did not correlate with a current infectious chlamydia load, as determined by PCR, suggesting that these animals had been previously naturally infected with Chlamydia and still contained residual anti-chlamydial antibodies. The presence of these antibodies did not appear to prevent either vaccine from triggering an immune response in vaccinated koalas. However, the vaccines showed a difference in their outcome regarding clearing of existing infections and protection against new ones. While the effect of our PmpG vaccine on clearance is still unknown, as we had no infected koalas at the time of vaccination in that cohort, it failed to prevent new infections by 6 and 9 months post-vaccination (5 koalas out of 21 developing a new infection). This suggests that, unfortunately, although immunogenic, the PmpG vaccine did not elicit the correct type or strength of response required for ongoing protection from new infections under natural conditions. This result is a little surprising as, in other chlamydial species, Pmp proteins have been described as dominant antigenic targets for T-cell immune responses, and have been reported to be immunogenic and protective against chlamydial reinfections in mice [14, 27, 28, 41, 42]. The selection of only one type of PmpG protein from one *C*. *pecorum* strain included in the vaccine might be an explanation. Indeed, the PmpG genes that are under positive selection in a specific strain are the most likely to encode proteins with antigenic regions capable of eliciting an immune response to a broader range of strains when included in a vaccine [43]. We previously identified nine PmpG genes in the *C*. *pecorum* genome [44, 45]. Amongst these nine PmpG genes, PmpG1 and 9 are under positive selection with pmpG1 having the highest polymorphism [31]. Although we chose PmpG1 for this vaccine, it is possible that PmpG9 or a combination of both would have been more potent in protecting against new infections. Importantly, recent vaccine trials have combined several Pmps of variable polymorphism to give rise to efficient vaccines [27, 28, 42]. This suggests that selecting and including several immunodominant *C*. *pecorum* PmpG proteins could be an option to provide a range of epitopes for both T cell recognition and antigen presentation in our koala population with variable MHC backgrounds, and further allow development of an adaptive immune response.

The 3MOMP vaccine clearly demonstrated a level of protection against current infection and also some protection initially, at least, against new infections as evidenced by the observation of no new infections in the first 6 months post-vaccination compared to the non-vaccinated control group. However, two koalas developed new infections between 6 and 9 months post-vaccination. This suggests that the adaptive immune response induced by the 3MOMP vaccine is not long lasting or might be too specific for some *C*. *pecorum* strains. This suggests a need to introduce more divergence in the vaccine epitopes in the future by selecting a larger number of MOMP proteins with multiple strain variations.

Systemic and mucosal B and T cell-mediated immunity have been shown to be necessary to elicit the adaptive immune response and provide protection against *C*. *trachomatis* infections particularly in the mouse model [46–49]. In line with these findings, in addition to inducing a *C*. *pecorum*-specific systemic immunity, vaccination of wild koalas with 3MOMP and PmpG vaccines triggered mucosal immunity. However, the EPT values for anti-chlamydial IgA and IgG were quite low in both UGT and ocular samples, for both vaccines. If such an immune response was sufficient to clear *C*. *pecorum* infections such as in the 3MOMP vaccine, they appear insufficient in the case of the PmpG vaccine. The route of immunization can determine tissue-tropism of the immune cells and generate immune responses in various tissues (48). Combined routes of immunization via intramuscular and intranasal delivery can further induce systemic and mucosal immunity in minipigs infected with *C*. *trachomatis* [49]. The adjuvant used in our study has been shown to be effective in intranasal delivery [50]. Therefore, a double immunization of koalas via muscular and mucosal routes (nasal or genital) might be the solution to boost the vaccine effect we already observed at the mucosal site by eliciting a stronger, and potentially longer lasting, immune response and triggering mucosal resident B and T cell-mediated immune responses.

All 3MOMP vaccinated koalas infected with *C*. *pecorum* at the time of vaccination cleared their infection by 6 months post-vaccination. In contrast, none of the non-vaccinated infected koalas naturally cleared their infection, with some koalas even further progressing to disease. It appears that this multi-epitope vaccine could be suitable to trigger the adaptive immune system and provide cross-protection against antigenic variants required to clear *C*. *pecorum* infections in our wild population. Our data analysis suggested that production of IgA might be associated with clearance of natural infections. The role of IgA in chlamydial infections remains controversial. Indeed, while the absence of IgA in IgA-deficient mice seems to have no effect on clearance of primary or secondary infections, other studies have reported the effect of anti-MOMP IgA (from either vaccination with a MOMP vaccine or adoptive transfer) on reducing chlamydial infections in mice [46, 47, 51]. Thus, although our data supported a role for IgA in lowering chlamydial burden in koalas, further experiments are required to fully elucidate the role of mucosal immunity in chlamydial infections.

Alongside humoral immunity, cell-mediated immunity is essential to fight chlamydial infection. The importance in IFN-γ produced by CD4 T cells for protective immunity against chlamydial infection has been demonstrated previously for *C*. *trachomatis* and *C*. *muridarum* [7]. In *C*. *pecorum*, previous experiments showed a strong expression of IFN-y in koalas with chlamydial infections, suggesting the same cytokine mechanisms as other chlamydial species [36]. In addition, the Th17 cells contribute to host protective immunity against bacterial pathogens and IL-17 secreted from Th17 cells was associated with protection against *C*. *muridarum* [52, 53]. Both 3MOMP and PmpG vaccines elicited a significantly increased expression of both IFN-γ and IL-17, post-vaccination.

Mechanisms of protection against chlamydia infections after vaccination have been investigated in other members of the genus. Indeed, recent experiments highlighting the role of cell-mediated immunity and IFN-γ production on protecting against chlamydial infection post-vaccinations. Although the role of IFN-γ has been well studied for *C*. *trachomatis*, and three mechanisms proposed including the up-regulation of nitric oxide synthase, the down regulation of the transferrin receptors for iron transport, or the inhibition of indoleamine 2,3-dioxygenase, these mechanisms have not yet been analysed in for animal *C*. *pecorum* infections [54]. Similarly, recent work on mice immunized with a vaccine containing a MOMP chimeric protein exhibiting selected T-cell and B-cell epitopes reduced shedding and immunopathology associated with production of IFN-γ, IL-17 and IL-13 [42]. Production of TNF-α has also been shown to participate in the clearance of chlamydial infection, potentially via up regulation of IFN-β, NK cells or neutrophils [55]. Our results here with *C*.*pecorum* infection in koalas confirms that production of IFN-γ plays a role in clearance and protection. In addition, IgA has also been suggested to be involved in protection against chlamydia. It has recently been reported that vaccinated minipigs produced a strong genital secretory IgA response and this correlated with protection against a live challenge in this model [49]. Again, our data here for the koala model suggest that mucosal IgA levels do correlate with protection. At this stage, we are unsure of the relative importance of these two immune mechanisms for protection in the koala / *C*.*pecorum* natural situation.

In our koala cohorts, we identified eight *C*. *pecorum ompA* haplotypes from 10 *C*. *pecorum* samples from infected koalas. Among these eight haplotypes, six were phylogenetically related to genotype F’ and two were identical to *C*. *pecorum* strains previously identified in sheep, cattle and koalas [31, 40]. Importantly, all the variant strains exhibited mutations resulting in non-synonymous amino acid changes located mostly in the variable domain 4 (VD4) of the resulting MOMP protein. As the VD4 domain is considered the most immunogenic domain in the MOMP protein [39, 56], we hypothesize that non-synonymous changes in the amino-acid content of this domain would lead to modifications of the overall structure of the MOMP protein, inducing changes in the structural epitopes. As a consequence, the repertoire of antibodies established with the 3MOMP vaccine might have a limited recognition of the variants of MOMP proteins expressed on *C*. *pecorum* naturally infecting strains in our koala population, ultimately reducing the protective effects of the vaccine.

In conclusion, we have shown that recombinant PmpG protein is immunogenic, similar to recombinant MOMP, and can elicit systemic and mucosal humoral and cell-mediated immune responses with production of specific anti-chlamydial IgG and IgA antibodies. In addition, the 3MOMP vaccine showed clearance of infection in six koalas suggesting that an appropriate and adequate immune response can be elicited by vaccination. However, we also identified a few koalas (2/21) that developed chlamydial disease in the 3MOMP and PmpG vaccine cohorts, similar to the non-vaccinated group, after longer time periods (Refer to Table 1, right columns, for chlamydiosis status of each koala in the trial). This suggests that the vaccines were potentially short lasting. Analysis of the *C*. *pecorum* strains by *ompA* genotyping revealed a large genetic diversity of the *ompA* strains amongst infected koalas in our trial. This might account for the reduced efficiency of the vaccine as all strains exhibited non-synonymous mutations in the VD4 domain of *ompA*, known to contain T and B cell epitopes. Although the capability of both vaccines to stimulate an adaptive response and be protective needs to be fully evaluated, this work illustrates the necessity to combine epitopes most relevant to a large panel of variable strains with an efficient adjuvant.

## Supporting information

S1 Fig Schematic of the MOMP-A, MOMP-F, MOMP-G and PMpG proteins included in the 3MOMP and PmpG vaccines. Numbering indicates the amino acid number in the full length protein. His corresponds to the hexa-histidine tag that consists of 6 histidine residues located at the N-terminus of the recombinant MOMP and PmpG proteins.
(TIF)
