# Supplementary figures and images for: Immunization of a wild koala population with a recombinant Chlamydia pecorum Major Outer Membrane Protein (MOMP) or Polymorphic Membrane Protein (PMP) based vaccine: New insights into immune response, protection and clearance
Source: PLoS One. 2017 Jun 2;12(6):e0178786. doi: 10.1371/journal.pone.0178786 (PMC5456371; doi:10.1371/journal.pone.0178786)

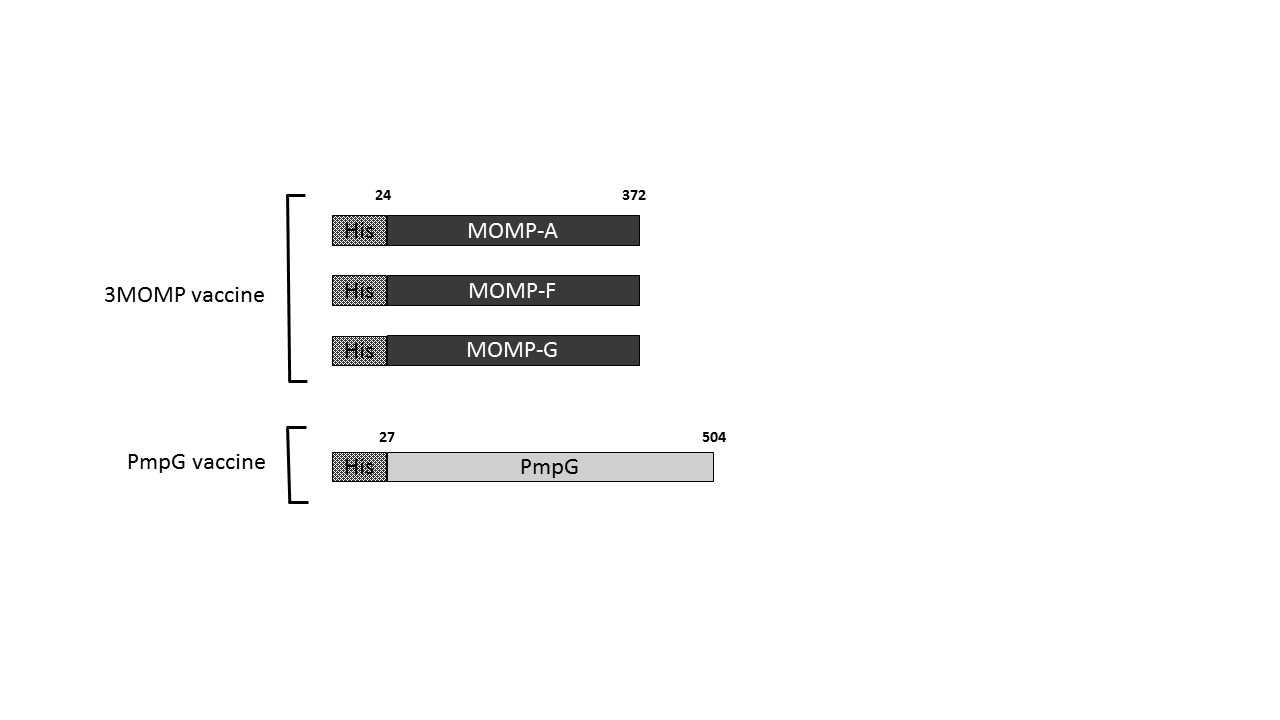

Supplement: S1 Fig — Numbering indicates the amino acid number in the full length protein. His corresponds to the hexa-histidine tag that consists of 6 histidine residues located at the N-terminus of the recombinant MOMP and PmpG proteins. (TIF) [file pone.0178786.s001.TIF]
